# Effect of magnesium supplementation in improving hyperandrogenism, hirsutism, and sleep quality in women with polycystic ovary syndrome: A randomized, placebo‐controlled clinical trial

**DOI:** 10.1002/hsr2.1013

**Published:** 2022-12-29

**Authors:** Mahsa Gholizadeh‐Moghaddam, Hatav Ghasemi‐Tehrani, Gholamreza Askari, Mahsima Jaripur, Cain C. T. Clark, Mohammad Hossein Rouhani

**Affiliations:** ^1^ Nutrition and Food Security Research Center, Department of Community Nutrition Isfahan University of Medical Sciences Isfahan Iran; ^2^ Department of Obstetrics and Gynecology Isfahan University of Medical Sciences Isfahan Iran; ^3^ Centre for Intelligent Healthcare Coventry University Coventry UK

**Keywords:** hirsutism, hyperandrogenism, magnesium, polycystic ovarian syndrome, sleep quality

## Abstract

**Background and Aims:**

Polycystic ovary syndrome (PCOS) is one of the common endocrine disorders in women, which causes numerous symptoms in women. The relationship of many micronutrients with this syndrome has been investigated. This study was conducted to examine the effects of magnesium supplementation on hyperandrogenism, hirsutism, and sleep quality in women with PCOS.

**Methods:**

In this parallel randomized clinical trial, 64 women with PCOS were randomly assigned to the magnesium group (*n* = 32) or placebo group (*n* = 32) for 10 weeks. Patients in the magnesium group received one 250 mg magnesium oxide tablet, per day. Hyperandrogenism, hirsutism, and sleep quality were measured at the beginning and end of the study. This randomized clinical trial was registered at https://www.IRCT.ir (IRCT20130903014551N8).

**Results:**

Magnesium supplementation had no significant effect on hyperandrogenism (*p* = 0.51 for dehydroepiandrosterone sulfates, *p* = 0.27 for testosterone), hirsutism (*p* = 0.23), and sleep quality (*p* = 0.85) compared with placebo.

**Conclusions:**

The present study showed that a single dose of magnesium supplementation elicited no beneficial effects on the mentioned symptoms in polycystic women. It is possible that the positive effects of magnesium observed in the former studies were due to the synergistic effects of other vitamins or minerals. More studies are needed in this area.

## 1 INTRODUCTION

Polycystic ovary syndrome (PCOS) is one of the most common endocrine disorders in women. The prevalence of PCOS is about 4%–21%, depending on the different diagnostic criteria.^1^ Women with PCOS have several symptoms including hirsutism, hyperandrogenism, and sleep disorders that affect the quality of life.^2^ However, it seems that some nutrients may have a role in the improvement of these conditions. Magnesium is a fundamental mineral in the human body, considered a common intracellular electrolyte,^3^ and is necessary for the function of 300 enzyme systems involved in energy metabolism and nucleic acid synthesis.^4^ Recent evidence has suggested that women with PCOS may have lower levels of serum magnesium compared with healthy women^5^, ^6^; in addition, it has been reported that magnesium may contribute to a reduction of PCOS symptoms. Some studies have shown that, in women with PCOS, serum magnesium concentration was higher in individuals without hirsutism compared with women with hirsutism.^7^ Also, an improvement in hirsutism was observed after cosupplementation of magnesium and vitamin E in women with PCOS.^8^ Serum magnesium levels may also be associated with hyperandrogenism^7^; indeed, a cohort study reported that insufficient dietary intake of magnesium was related to hyperandrogenism among women with PCOS.^9^ Similarly, there was an inverse association between serum magnesium and testosterone concentration among patients with PCOS.^10^ On the other hand, magnesium supplementation significantly reduced sleep disorders in the elderly,^11^ and a systematic review and meta‐analysis showed that oral magnesium supplementation may improve insomnia and sleep quality in older adults.^12^

There is a lack of enough evidence regarding magnesium supplementation in women with PCOS. Indeed, previous studies have not focused on the effect of magnesium supplementation on sleep quality and hyperandrogenism in these patients, and although some studies examined the impact of magnesium supplementation on hirsutism, such studies used magnesium in combination with other nutrients and did not evaluate the effect of magnesium alone on hirsutism in PCOS. Therefore, the aim of the present clinical trial was to evaluate the effect of magnesium supplementation on hirsutism, hyperandrogenism, and sleep disorders in women with PCOS.

## 2 METHODS

### 2.1 Participants and randomization

The present study was a parallel double‐blind randomized controlled trial, with a duration of 10 weeks, conducted between October 2020 to October 2021 in Isfahan, Iran. Women referred to Isfahan Shahid Beheshti's infertility clinic were assessed for inclusion and exclusion criteria. The inclusion criteria involved: (1) were between 18 and 45 years old; (2) had PCOS according to the Rotterdam criteria^13^; (3) did not change the dose of the medications or start taking a new medication during the preceding 2 weeks; (4) were not menopausal; and (5) did not use other vitamin and mineral supplements. Based on the Rotterdam criteria, women with two of the following three symptoms were diagnosed as having PCOS: (1) no ovulation or low ovulation; (2) increased levels of blood androgens; and (3) polycystic ovaries on ultrasound (at least 12 follicles in each ovary).^13^ Patients were excluded if they: (1) changed the dosage of existing medication or started taking a new medication during the study; (2) were pregnant during the study; and (3) were menopausal before or during the study.

The following equation was used to estimate the required sample size:

$$N=2[{\left(Z_{1–\alpha /2}+Z_{1-\beta }\right)}^{2}\times S^{2}]/\Delta^{2}=2[{\left(1.96+0.85\right)}^{2}\times {\left(0.19\right)}^{2}]/{\left(0.122\right)}^{2}=30.$$

In this equation, *α* = 0.05 and *β* = 0.20 (the power of the study was 80%). The serum concentration of dehydroepiandrosterone sulfate (DHEAS) was considered as the main variable. According to the previous studies, ∆ = 0.122 µmol/l and *S* ^2^ = 0.19 µmol/l.^14^ Therefore, the required sample size in each group was 30. Due to the possible withdrawals during the study, we enrolled 64 participants (*n* = 32 in each group). Included participants were randomly assigned in a ratio of 1:1 to either magnesium or placebo using a computer‐generated randomization sequence in permuted blocks of two, with individuals categorized according to the body mass index (≤24.9 and >24.9 kg/m^2^). Staff provided a randomization list and numbered supplement containers. All participants, care providers, and the analyzers of the outcomes were blinded to the study. All participants signed a written consent form, before study commencement. This study was ethically approved by The Research Council and Ethical Committee of Isfahan University of Medical Sciences, Isfahan, Iran (Code: IR.MUI.RESEARCH.REC.1399.404), and was registered at https://www.IRCT.ir (IRCT20130903014551N8).

### 2.2 Intervention

Patients received sufficient information regarding the study. Women in the magnesium group received a 250 mg magnesium oxide tablet (Magni One® produced by DonyaDarou) per day for 10 weeks. women in the placebo group received a tablet containing 5 mg starch, and its color, appearance, smell, and taste were similar to the magnesium tablet. Participants in both groups were recommended to take the tablets after breakfast. Participants were monitored through a telephone call and virtual networks, and they were asked to give back empty containers of supplements to ensure that all supplements were used properly.

A list of following dietary recommendations was provided for participants in both groups: (1) to avoid consuming refined or simple carbohydrates, such as white bread, white rice, sugar, and sweets; (2) consume higher amounts of fresh vegetables; (3) increase the number of meals and reduce the volume of each meal; (4) drink adequate fluid, especially water (at least eight glasses per day); (5) replace starchy vegetables, such as potatoes with nonstarchy vegetables, such as lettuce, cabbage, cucumber, and so on; one study has shown that consuming a low starch diet such as low starch carbohydrates resulted in weight loss, improved insulin sensitivity, and reduced testosterone in women with PCOS^15^; (6) consume fresh fruits instead of industrial and sugar‐sweetened fruit juices; (7) avoid consuming salty foods, fast foods, and high‐fat dairy products; and (8) consume healthy oils, such as olive oil and canola oil, instead of saturated or partially saturated vegetable oil, or animal fat.

### 2.3 Biochemical assessment

The serum concentration of magnesium, DHEAs, and testosterone was measured at baseline, and DHEAs and testosterone were remeasured after 10 weeks. A 10 ml blood sample was drawn, and after serum isolation, magnesium was measured by the atomic absorption spectrophotometry method. Also, radioimmunoassay and high‐performance liquid chromatography were used to measure the level of testosterone and DHEAs, respectively.

### 2.4 Assessment of sleep quality

Sleep quality was assessed using the Pittsburgh Sleep Quality Questionnaire (PSQI) completed by a trained staff. The validity and reliability of this questionnaire were evaluated and deemed to be acceptable.^16^, ^17^ This questionnaire examines the quality of sleep over the past month and includes 19 questions in 7 domains (subjective sleep quality, sleep latency, sleep duration, habitual sleep efficiency, sleep disturbances, use of sleeping medication, daytime dysfunction). Each domain was scored from 0 to 3 points, and the total score of the PSQI sleep quality questionnaire ranged from 0 to 21, where higher scores indicated lower sleep quality. A score greater than 5 indicates that the person was sleeping less.^16^

### 2.5 Assessment of hirsutism

A modified Ferriman–Gallwey questionnaire was used to assess the hirsutism status and was completed by a trained staff. The validity and reliability of this questionnaire were previously evaluated and shown to be acceptable.^18^ In this questionnaire, the density of hairs at three sites (upper lip, thigh, and lower abdomen) were measured. A score of 0–4 was given based on the density of the terminal hairs. A score higher than 4 indicates the presence of hirsutism.

### 2.6 Assessment of dietary intake and physical activity

To control the confounding effect of dietary intake and physical activity, five 1‐day food diaries and five 1‐day physical activity records were completed by each participant during the study. Records covered three usual days and two weekend days. Patients were visited at baseline, the 5th week, and at the end of the study. Food diaries and physical activity records were provided during these visits. Food records were analyzed using Nutritionist IV designed based on the USDA food composition database. Individuals were asked to report their activities, including walking, exercise, sleep, hours spent watching TV, housework, studying, bathing, and other activities, and subsequently, the total metabolic equivalent (MET) was calculated by multiplying the frequency, duration, and intensity of each physical activity in 24 h.^19^ Physical activity was reported in terms of MET per hour (MET/h/day).

### 2.7 Statistical analysis

An intention‐to‐treat (ITT) analysis based on the linear regression method was applied in the present study.^20^ Normal distribution was evaluated by using the Kolmogorov–Smirnov test and visual inspection of Q–Q plots, and we found that the distribution of DHEAs was not normal. Therefore, a log transformation method was used to attenuate non‐normal distribution. We compared qualitative variables between the magnesium and placebo groups using the *χ* ^2^‐test. Categorical variables were reported as percentages. Baseline and endpoint values were compared within each group by paired *t*‐test analysis. Intergroup comparisons were performed by independent Student's *t*‐test for numerical variables. Confounding variables (energy intake and baseline values) were adjusted by analysis of covariance. Numerical variables were reported as mean ± SD, except for log‐transformed variable (i.e., DHEA), which was reported as geometric mean ± SD. All data analyses were conducted using Statistical Package for the Social Sciences (SPSS) version 21 statistical software from IBM company, with an a priori *α*‐level of 0.05. We used two‐sided tests for all variables.

## 3 RESULTS

**Figure 1:**
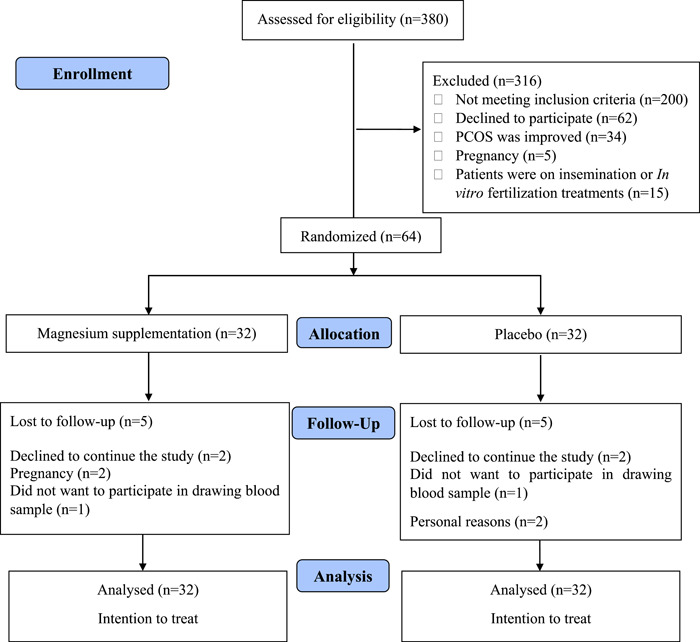
Study procedure based on the CONSORT flow diagram

Figure 1 shows the flowchart of the intervention. Data of 380 patients referred to the infertility clinic were screened, and 316 patients were excluded because of the following reasons: (1) individuals did not meet the inclusion criteria for the study (*n* = 200; 0.2) patients declined to participate in the study (*n* = 62); (3) PCOS was treated (*n* = 34); (4) women were pregnant (*n* = 5); and (5) patients were on insemination or in vitro fertilization treatments (*n* = 15). Therefore, 64 patients were included in the study and were randomly assigned to the magnesium (*n* = 32) or placebo (*n* = 32) groups. Five patients were lost to follow‐up in the magnesium group because they: (1) declined to continue the study; (2) became pregnant; or (3) were not comfortable providing a blood sample. Similarly, five individuals were lost to follow‐up in the placebo group because (1) they declined to continue the study; (2) were not comfortable providing a blood sample; or (3) for personal reasons. Therefore, 54 patients completed the study. By using an ITT method, data from 64 participants (*n* = 32 in each group) were included in statistical analysis.

The general characteristics of the participants are shown in Table 1. Accordingly, results revealed that age (*p* = 0.62), weight (*p* = 0.92), height (*p* = 0.44), body mass index (*p* = 0.81), overweight or obesity (*p* = 0.99), educational status (*p* = 0.38), economic status (*p* = 0.17), marital status (*p* = 0.31), and the level of physical activity (*p* = 0.73) were not significantly different between two groups. However, baseline serum magnesium levels were significantly higher in the magnesium group than in the placebo group (*p* = 0.04).

**Table 1:** General characteristics of the participants

Variable	Magnesium (*n* = 32)	Placebo (*n* = 32)	*p* Value
Age (years)	31.69 ± 5.41	32.44 ± 6.42	0.62
Weight (kg)	69.88 ± 14.36	70.22 ± 12.22	0.92
Height (m)	1.6 ± 0.07	1.62 ± 0.05	0.44
BMI (kg/m^2^)	26.89 ± 4.68	26.63 ± 4.06	0.81
Overweight/obese (%)	22 (68.5%)	22 (68.7%)	0.99
Education (%)			
Did not complete high school	8 (25%)	5 (15.6)	
High school	15 (46.9%)	13 (40.6%)	0.38
University degree	9 (28.1%)	14 (43.8%)	
Economic status (%)			
Low	6 (18.8%)	3 (9.4%)	
Medium	23 (71.9)	21 (65.6%)	0.17
High	3 (9.4%)	8 (25%)	
Married (%)			
No	3 (9.4%)	1 (3.1%)	0.31
Yes	29 (90.6%)	31 (96.9%)	
Physical activity (MET/h)	1.1 ± 0.13	1.11 ± 0.17	0.73
Serum magnesium (mg/dl)	2.35 ± 0.21	2.25 ± 0.17	0.04

Abbreviations: BMI, body mass index; MET, metabolic equivalent.

Table 2 shows the energy‐adjusted dietary intake of participants during the study. There were no differences in dietary intake between the two groups.

**Table 2:** Energy‐adjusted dietary intake of participants during the study

Nutrients		Magnesium (*n* = 32)	Placebo (*n* = 32)	*p* Value
Carbohydrate (g/day)		231.819 ± 91.16	217.082 ± 89.45	0.52
Protein (g/day)		59.925 ± 9.55	59.41 ± 9.37	0.83
Fat (g/day)		95.74 ± 21.11	92.07 ± 20.72	0.49
Saturated fatty acids (g/day)		19.78 ± 7.89	18.52 ± 7.74	0.52
Monounsaturated fatty acids (g/day)		26.54 ± 12.84	23.72 ± 12.61	0.38
Polyunsaturated fatty acids (g/day)		41.51 ± 14.27	39.98 ± 14.01	0.67
Linoleic acid (g/day)		39.85 ± 13.86	37.41 ± 13.6	0.48
Cholesterol (mg/day)		194.13 ± 151.61	267.97 ± 148.78	0.06
Vitamin A (Retinol equivalent/day)		537.10 ± 977.60	739.02 ± 959.31	0.41
Vitamin E (mg/day)		3.30 ± 3.1	3.12 ± 3.05	0.82
Vitamin K (μg/day)		70.75 ± 64.87	80.53 ± 63.65	0.55
Vitamin C (mg/day)		121.03 ± 80.56	100.55 ± 79.05	0.31
Vitamin B1 (mg/day)		1.51 ± 0.36	1.44 ± 0.36	0.42
Vitamin B2 (mg/day)		1.33 ± 1.11	1.17 ± 1.09	0.57
Vitamin B3 (mg/day)		19.92 ± 5.65	19 ± 5.54	0.52
Vitamin B5 (mg/day)		4.18 ± 1.62	3.77 ± 1.58	0.31
Vitamin B6 (mg/day)		1.21 ± 0.66	1.43 ± 0.64	0.18
Folate (µg/day)		215.68 ± 99.55	211.28 ± 97.69	0.86
Sodium (mg/day)		942.93 ± 417.02	1039.27 ± 409.22	0.36
Potassium (mg/day)		1959.2 ± 469.97	1829.8 ± 461.18	0.27
Calcium (mg/day)		489.73 ± 167.72	502.43 ± 164.58	0.76
Zinc (mg/day)		6.53 ± 1.97	6.24 ± 1.93	0.56
magnesium (mg/day)		176.20 ± 56.30	169.26 ± 55.24	0.62
Iron (mg/day)		15.52 ± 5.42	16.48 ± 5.32	0.48
Dietary fiber (g/day)		13.30 ± 5.97	12.87 ± 5.86	0.77

*Note*: Variables are expressed as mean ± SD. All variables were adjusted for total energy intake.

The effects of magnesium supplementation on hyperandrogenism, hirsutism, and sleep quality are shown in Table 3. DHEAs (*p* ^b^ < 0.001 in the magnesium group and *p* ^b^ < 0.001 in the placebo group) increased significantly at the end of the trial compared with the baseline in both groups. Testosterone concentration was higher in the placebo group at the end of the study compared to the baseline (*p* ^b^ = 0.01). Contrary to our expectation, the hirsutism score decreased in the placebo group at the end of the trial (*p* ^b^ = 0.03). The quality of sleep did not show any significant change in any of the groups at the end of the study (*p* value (*p* ^b^) for the magnesium group = 0.61, *p* value (*p* ^b^) for the placebo group = 0.74). Comparison of measurements at the end of the study between the two groups did not show significant differences in terms of testosterone (*p* ^c^ = 0.20), DHEAs (*p* ^c^ = 0.16), hirsutism (*p* ^c^ = 0.42), and sleep quality (*p* ^c^ = 0.73). These findings did not change after adjusting the baseline values of each factor and serum magnesium (*p* ^d^ = 0.27 for testosterone, *p* ^d^ = 0.51 for DHEAs, *p* ^d^ = 0.23 for hirsutism, and *p* ^d^ = 0.85 for quality sleep).^1^

**Table 3:** Effects of magnesium supplementation on hyperandrogenism, hirsutism, and sleep quality^a^

Variables	Magnesium (*n* = 32)				Placebo (*n* = 32)					*p* Value^c^	*p* Value^d^
	Baseline	End of trial	Change	*p* Value^b^	Baseline	End of trial	Change	*p* Value^b^	95% CI		
Testosterone (pg/ml)	1.35 ± 0.21	1.39 ± 0.44	0.03 ± 0.98	0.83	1.13 ± 0.62	1.56 ± 0.60	0.43 ± 0.87	0.01	−0.118, 0.419	0.20	0.27
DHEA (μg/dl)	142.80 ± 2.29	193.36 ± 2.01	46.13 ± 93.02	<0.001	168.74 ± 1.58	240.35 ± 1.65	85.35 ± 101.78	<0.001	−16.03, 84.03	0.16	0.51
Score of hirsutism	4.97 ± 2.87	4.53 ± 3.35	−0.43 ± 2.92	0.41	4.94 ± 2.60	3.96 ± 1.97	−0.96 ± 2.45	0.03	−1.96, 0.49	0.42	0.23
Score of sleep quality	5.71 ± 3.52	5.96 ± 2.78	0.25 ± 2.77	0.61	6.06 ± 2.95	6.25 ± 3.52	0.18 ± 3.17	0.74	−1.48, 1.23	0.73	0.85

Abbreviations: CI, confidence interval; DHEA, dehydroepiandrosterone.

^a^ Variables are expressed as mean ± SD except for DHEA reported as geometric mean ± SD.

^b^ Obtained from paired *t*‐test comparing baseline and endpoint values within each group.

^c^ Obtained from an independent *t*‐test comparing endpoint measurements between two groups.

^d^ Obtained from analysis of covariance, adjusted for baseline value of each factor and baseline serum magnesium comparing endpoint values between two groups.

## 4 DISCUSSION

Results of this randomized clinical trial showed that supplementation with 250 mg/day of magnesium did not significantly improve hyperandrogenism, hirsutism, and sleep quality in women with PCOS. Consumption of dietary supplements has been increasing globally^21^; however, several supplements have no beneficial effect on health and might have long‐term negative side effects.^22^ Therefore, it would be advantageous for studies to evaluate the efficacy of dietary supplements in different diseases, such as PCOS.

In the present study, we found that magnesium supplementation had no significant effect on hyperandrogenism and hirsutism. Although previous studies have reported that magnesium may have beneficial effects on hyperandrogenism and hirsutism, such studies have used magnesium in combination with other nutrients. Indeed, a clinical trial reported that cosupplementation of magnesium and vitamin E may improve hormonal status and hirsutism in patients with PCOS.^8^ Another intervention study showed that cosupplementation of magnesium, zinc, calcium, and vitamin D regulated hormonal profile and biomarkers of inflammation and oxidative stress in women with PCOS.^23^

The results of the present study revealed that magnesium supplementation could not change sleep quality. Although a clinical trial reported an improvement in insomnia following magnesium supplementation, magnesium was used in combination with melatonin and vitamin B complex.^24^ Moreover, the results of an observational study showed no associations between dietary magnesium intake and daytime sleepiness nor night snoring in men and women.^25^ A systematic review summarized the effect of oral magnesium supplementation on insomnia in older adults and reported that there was insufficient evidence to advocate the prescription of magnesium as a complementary therapy for insomnia.^12^

As mentioned, the present study did not observe a significant effect of magnesium supplementation on the improvement of hyperandrogenism, hirsutism, and sleep quality in women with PCOS. Some possible reasons for these nonsignificant findings are: (1) Most studies that reported a significant effect of magnesium supplementation used cosupplementation of magnesium and other nutrients. Therefore, it seems that synergistic interaction between nutrients may have contributed to beneficial effects. For example, vitamin E and magnesium are known to have a synergistic interaction and strengthen each other in biological pathways.^26^, ^27^ Also, vitamin B6 has synergistic effects on magnesium function,^28^ and the synergistic interaction between vitamin C and vitamin B12 and magnesium, respectively has been approved.^29^, ^30^ Therefore, magnesium may be more effective when used in combination with other nutrients. (2) Participants of our study were not magnesium deficient; where the mean baseline serum magnesium concentration was 2.3 mg/dl among participants and the normal range of serum magnesium in adults is 1.7–2.3 mg/dl. However, it seems that the positive effects of magnesium supplementation are more likely to be observed in individuals with magnesium deficiency. (3) We used a 250 mg magnesium supplementation; however, higher doses of magnesium may confer beneficial effects in the short term. A previous study that used 607 mg/day reported favorable effects on systolic and diastolic blood pressure,^31^ while, in another study, magnesium supplementation, 350 mg/day, improved insulin resistance in patients with nonalcoholic fatty liver disease.^32^ In the present study, we used 250 mg/day of magnesium because the evidence for prescribing higher doses in women with PCOS is limited.

Magnesium absorption is not fast with ≈80% of ingested magnesium being absorbed within 6–7 h.^33^ Moreover, previous studies suggested that magnesium may affect sleep quality via long‐term mechanisms such as renin–angiotensin–aldosterone system and hypothalamic‐pituitary‐adrenal axis, depressive symptoms, and anemia^25^, ^34^ Therefore, it is unlikely that magnesium has a postprandial effect on sleep quality. Although there is no reliable source that has clearly stated when is the best time of day for taking a magnesium supplement, it should be consumed after a meal. Therefore, we decided to prescribe it after breakfast because participants were not fast.

In the present study, some strengths and limitations should be acknowledged. Indeed, first and foremost, the Covid‐19 pandemic and subsequent lockdown had an adverse effect on the follow‐up of the patients. In addition, we utilized subjective data collection methods for some variables. Another limitation of the present study is the use of magnesium oxide despite its lower absorption in comparison with other types. Since we needed pure magnesium, a supplement without any other vitamins or minerals, for use in the study, the only available supplement, was magnesium oxide. Other magnesium compounds such as magnesium glycinate were mixed with other nutrients. Nevertheless, despite the limitations of the present work, a strength of this study is that we implemented an ITT method in statistical analysis and utilized well‐validated data collection methods. Furthermore, in instances of subjective data collection methods, these questionnaires/surveys were completed with the support of trained staff.

In conclusion, 250 mg/day of magnesium supplement did not significantly improve hyperandrogenism, hirsutism, and sleep quality disorders in women with PCOS, and unnecessary supplementation should be avoided.

## AUTHOR CONTRIBUTIONS

**Mahsa Gholizadeh‐Moghaddam**: Data curation; investigation; software; writing – original draft; writing – review and editing. **Hatav Ghasemi‐Tehrani**: Conceptualization; data curation; methodology; resources; software; validation; writing – original draft. **Gholamreza Askari**: Conceptualization; funding acquisition; resources; validation; visualization; writing – original draft. **Mahsima Jaripur**: Data curation; investigation; methodology; software; writing – original draft. **Cain C. T. Clark**: Resources; validation; visualization; writing – original draft; writing – review and editing. **Mohammad Hossein Rouhani**: Conceptualization; data curation; formal analysis; methodology; project administration; writing – original draft. All authors have read and approved the final version of the manuscript. M. H. R. as the corresponding author had full access to all of the data in this study and takes complete responsibility for the integrity of the data and the accuracy of the data analysis.

## CONFLICT OF INTEREST

The authors declare no conflict of interest.

## ETHICS STATEMENT

This study was ethically approved by The Research Council and Ethical Committee of Isfahan University of Medical Sciences, Isfahan, Iran, (Code: IR.MUI.RESEARCH.REC.1399.404), and was registered at IRCT.ir on 2020‐10‐05 (Registration Code: IRCT20130903014551N8). Also, all participants completed an informed consent form.

## TRANSPARENCY STATEMENT

Mohammed Hossein Rouhani as the lead author affirms that this manuscript is an honest, accurate, and transparent account of the study being reported. The reporting of this work is compliant with CONSORT guidelines. The lead author affirms that no important aspects of the study have been omitted and that any discrepancies from the study as planned. The Research Council and Ethical Committee of Isfahan University of Medical Sciences, Isfahan, Iran and Food Security Research Center, Isfahan University of Medical Sciences, Isfahan, Iran approved this study (Code: IR.MUI.RESEARCH.REC.1399.404). This randomized clinical trial was registered at https://www.IRCT.ir on October 5, 2020 (Registration Code: IRCT20130903014551N8).

## Data Availability

Data are available on request from the authors.

## ENDNOTE

1 Superscript b indicate *p* value for within group comparison. Superscript c indicate *p* value for between group comparison. Superscript d indicate *p* value for between group comparison after adjusting for confounders.
